# CircadiOmics: bioinformatics platform for circadian rhythms

**DOI:** 10.1093/bioinformatics/btag716

**Published:** 2026-09-28

**Authors:** Kousha Changizi Ashtiani, Pierre Baldi

**Affiliations:** Artificial Intelligence in Science Institute, University of California, Irvine, CA 92697, United States; Department of Computer Science, University of California, Irvine, CA 92697, United States; Artificial Intelligence in Science Institute, University of California, Irvine, CA 92697, United States; Department of Computer Science, University of California, Irvine, CA 92697, United States

## Abstract

**Motivation:**

Circadian rhythms are fundamental to biology: they occur at the molecular level across all species and are critical to homeostasis and biological functions. They are also essential for health and medicine, as disruption of circadian regulation has been linked to numerous health problems, and many drug targets exhibit circadian oscillations, highlighting the importance of molecular-level circadian insight for precision medicine. Despite the rapid growth of circadian omic time series datasets, informatics tools for processing and analyzing this growing wealth of data remain limited.

**Results:**

CircadiOmics provides, to the best of our knowledge, the largest repository and analytical platform for circadian omic data, consisting primarily of transcriptomic data, along with metabolomic, proteomic, and acetylomic datasets. CircadiOmics contains 704 omic datasets across 18 species, 30 tissue/organ categories, and 21 experiment types, including genetic manipulations, dietary interventions, and disease-associated conditions. By leveraging this repository, users can access an environment for comparing and visualizing molecular-level circadian oscillations. Periodicity statistics computed using BIO_CYCLE, including period, amplitude, phase, *p*-values, and *q*-values, are available as interactive plots and downloadable tables. This updated version of CircadiOmics introduces new features and substantially expanded data coverage, establishing it as a comprehensive resource for the exploration and analysis of circadian omic data.

**Availability and implementation:**

CircadiOmics is freely accessible at: https://circadiomics.igb.uci.edu. The source code for BIO_CYCLE is available on GitHub: https://github.com/BaldiLab/BIO_CYCLE and archived on Zenodo: https://doi.org/10.5281/zenodo.22108329.

## 1 Introduction

Circadian rhythms date back to the origin of life and are found in species ranging from cyanobacteria to mammals (Panda *et al*. 2002b, Vijayan *et al*. 2009). They coordinate fundamental biological processes, including the sleep-wake cycle, metabolism, and cognitive processes (Takahashi *et al*. 2008, Gerstner *et al*. 2009). Disruption of circadian rhythms has been linked to a range of health problems, including depression, learning deficits, cardiovascular disease, diabetes, obesity, cancer, and premature aging (Knutsson 2003, Kondratov *et al*. 2006, Takahashi *et al*. 2008, Gerstner *et al*. 2009, Froy 2010). In addition, a substantial fraction of known drug targets exhibit circadian oscillations in one or more tissues, suggesting that the timing of therapeutic interventions can significantly influence drug efficacy and toxicity (Zhang *et al*. 2014).

Advances in high-throughput technologies have enabled the study of circadian rhythms at the molecular level. In circadian omic experiments, molecular species are measured over time, typically across 24–48 hours and with multiple biological replicates collected at each time point. Such studies have revealed that circadian oscillations are widespread and coordinated across molecular systems (Eckel-Mahan *et al*. 2012, Patel *et al*. 2015).

At the transcriptomic level, these oscillations arise from transcription-translation feedback loops regulated in part by the core clock, which comprises approximately a dozen key genes, including *CLOCK*, *BMAL1*, *PER1*, *PER2*, *CRY1*, and *CRY2* (Ko and Takahashi 2006, Masri *et al*. 2013, Patel *et al*. 2015). Although the core clock is composed of only a small number of genes, around 10% of transcripts and metabolites exhibit circadian oscillations in a given experiment (Storch *et al*. 2002, Panda *et al*. 2002a). Yet the set of oscillating molecular species varies substantially across tissues and under genetic, epigenetic, and environmental perturbations (Masri *et al*. 2014, Yao *et al*. 2025). This phenomenon is a major focus of ongoing research aimed at understanding how environmental factors, such as diet, drug treatments, and inflammation, reshape circadian molecular landscapes and how these oscillations are coordinated across tissues (Storch *et al*. 2002, Panda *et al*. 2002a, Bellet *et al*. 2013, Eckel-Mahan *et al*. 2013, Haspel *et al*. 2014, Murakami *et al*. 2016, Dyar *et al*. 2018, Torimitsu *et al*. 2026).

As circadian omic datasets continue to accumulate, both the scale and diversity of available data have expanded substantially, increasing the need for informatics resources that support comparative analysis across experiments. CircadiOmics was originally developed to provide a centralized platform for accessing and analyzing circadian omic time series data and has since been continuously expanded to reflect the field’s rapid growth (Ceglia *et al*. 2018, Samad *et al*. 2022). In this updated release, CircadiOmics has been substantially expanded in dataset coverage, species and tissue representation, and analytical scope, including the addition of a dedicated section for single-cell circadian analysis. Through these advances, CircadiOmics provides a platform for browsing, analyzing, and comparing circadian omic data across experiments (Fig. 1A). CircadiOmics is freely available at https://circadiomics.igb.uci.edu, with no login requirement.

**Figure 1:**
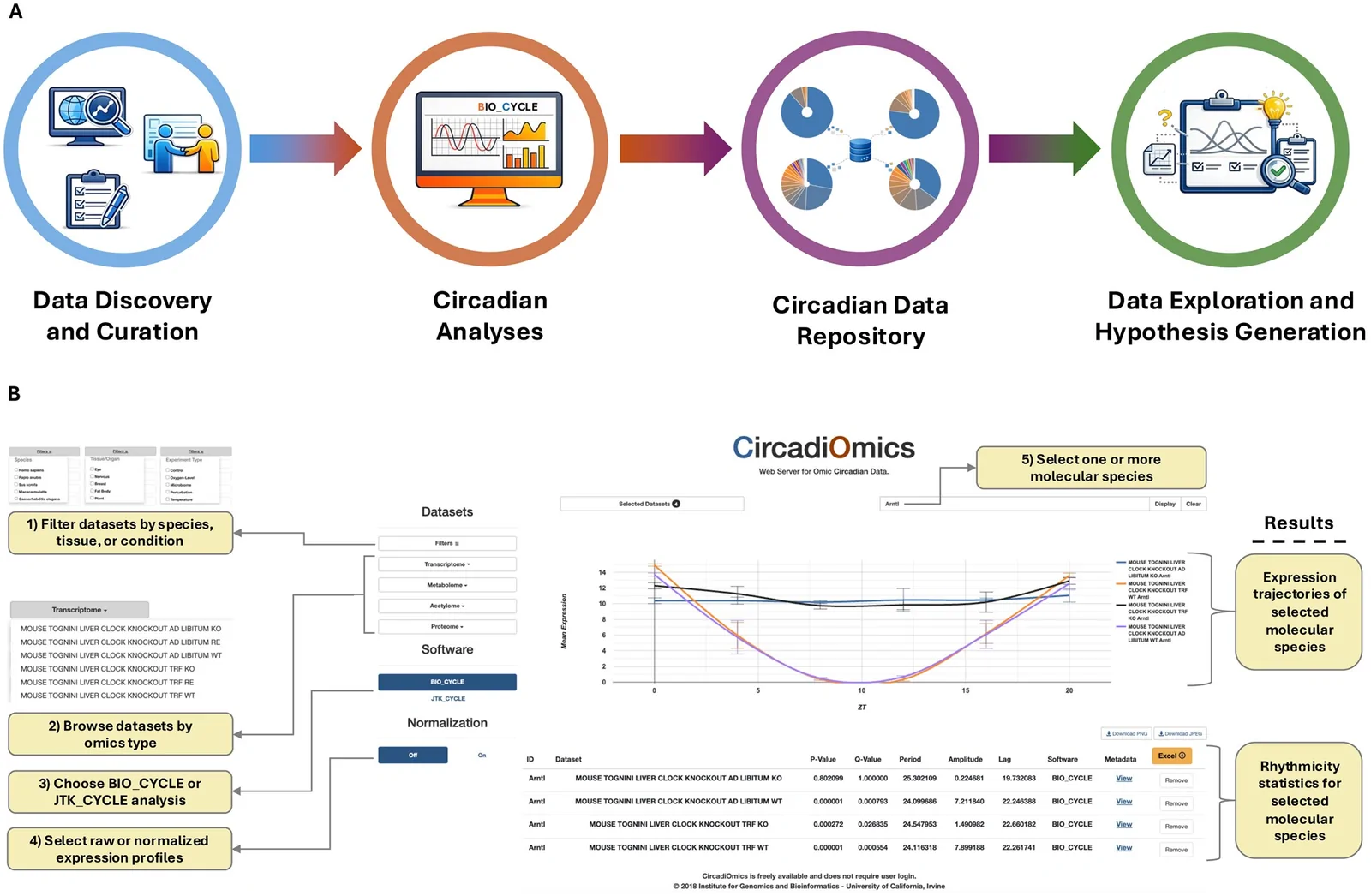
(A) Workflow of CircadiOmics, including dataset discovery and curation, circadian analyses, and repository integration, which together enable biological data exploration and hypothesis generation. **(B)** Overview of the CircadiOmics search and visualization tool and its step-by-step workflow. An example use case is shown in which CircadiOmics provides expression trajectories and rhythmicity statistics for *Arntl* (*Bmal1*), a core clock gene, within a single study across different conditions.

## 2 Materials and methods

### 2.1 Dataset coverage

Since the previous release, CircadiOmics has more than doubled in size. At the time of this publication, its repository includes 704 omic datasets associated with 18 species and more than 100 tissues, as reported in the source studies; these tissues are organized in CircadiOmics into 30 tissue/organ categories (Table S1, available as supplementary data at *Bioinformatics* online). Compared with several prominent circadian data repositories (Pizarro *et al*. 2013, Li *et al*. 2018, Castellana *et al*. 2022, Zhu *et al*. 2025), CircadiOmics contains substantially more datasets and provides the broadest tissue coverage, while covering only one fewer species than the repository with the greatest species coverage (Table S2, available as supplementary data at *Bioinformatics* online).

The CircadiOmics repository spans multiple omic modalities. Transcriptomic datasets constitute the majority of entries, but the repository also includes metabolomic, proteomic, and acetylomic datasets (Fig. S1A, available as supplementary data at *Bioinformatics* online), enabling analysis of circadian regulation within each represented omic modality and across diverse biological contexts. CircadiOmics also provides strong representation across commonly studied species, including *Mus musculus*, *Papio anubis*, *Homo sapiens*, *Drosophila melanogaster*, and *Rattus norvegicus* (Fig. S1B, available as supplementary data at *Bioinformatics* online). At the tissue/organ level, extensive sampling is available for widely studied systems such as liver, brain, muscle, heart, and skin (Fig. S1C, available as supplementary data at *Bioinformatics* online). In addition, CircadiOmics captures broad experimental diversity across 21 categories, including genetic manipulations, dietary interventions, disease-associated conditions, pharmacological treatments, and exercise paradigms (Fig. S1D, available as supplementary data at *Bioinformatics* online). These categories are further resolved into more than 90 associated conditions, providing a more detailed view of the experimental settings represented across the dataset collection (Table S3, available as supplementary data at *Bioinformatics* online). Together, these data enable exploration of circadian regulation across a wide range of physiological and pathological contexts.

### 2.2 Data collection

Datasets included in CircadiOmics are collected through a continuous discovery and curation process. Newly available circadian datasets are primarily identified using two automated discovery systems: an in-house web crawler and the publicly available monitoring service PubCrawler (Hokamp and Wolfe 2004). The in-house web crawler leverages the Python packages scholarly and geotools to scan the literature for recently published circadian omic studies and their associated datasets. Datasets flagged by these automated methods are subsequently reviewed and curated manually to assess their relevance to circadian research and data quality. In addition to automated discovery, datasets are incorporated through direct collaborations with other laboratories and periodic manual reviews of newly published studies, enabling the repository to remain current by steadily expanding its dataset coverage.

For each selected dataset, CircadiOmics uses the data and molecular measurements provided by the original study or its associated public repository for each time point and replicate, without performing additional preprocessing. The corresponding dataset metadata and source publication are also retained and made available in CircadiOmics. Because datasets originate from different studies and may have been generated using different experimental and normalization procedures, absolute molecular measurements should generally not be interpreted as directly comparable across studies.

### 2.3 Circadian analyses

All datasets in CircadiOmics are processed with an updated version of BIO_CYCLE together with JTK_CYCLE to compute oscillation statistics for molecular species in each experiment, including period, amplitude, phase, offset, *P*-values and *q*-values for periodicity (Hughes *et al*. 2010, Agostinelli *et al*. 2016). BIO_CYCLE serves as the primary circadian-analysis engine in CircadiOmics and is based on a deep neural network (DNN) implemented using PyTorch, trained on both synthetic and biological circadian time series data.

Since the previous update, BIO_CYCLE has undergone several methodological changes, including (i) *P*-value estimation that incorporates replicate variability at each time point; (ii) support for uneven or partially missing sampling by training with masked time points; and (iii) the use of a DNN architecture with residual blocks (Fig. S2, available as supplementary data at *Bioinformatics* online). Details of these methodological updates are provided in Supplementary Note 1, available as supplementary data at *Bioinformatics* online.

### 2.4 Implementation

CircadiOmics is implemented as a three-tier web application following a Model–View–Controller design. The server is built with the Flask Python framework, and the user interface is generated dynamically using Bootstrap and Google Charts. The web interface has also been updated to improve accessibility in line with the Americans with Disabilities Act and to meet Web Content Accessibility Guidelines 2.1 Level AA standards. For efficient querying, dataset information is cached as JSON-serialized files during server startup to support fast query response times.

## 3 Platform features and use cases

### 3.1 Search and visualization

The main focus of CircadiOmics is its interactive search and visualization framework, which enables users to explore and compare oscillatory patterns of molecular species across circadian experiments. Users can query molecular species across multiple datasets, and the platform provides overlaid visualizations of expression trajectories. For each query, CircadiOmics reports key periodicity statistics, including period, amplitude, phase, offset, *P*-values, and *q*-values. These metrics can be computed using either BIO_CYCLE or JTK_CYCLE. To facilitate dataset access, users can also filter datasets by species, tissue/organ, and experiment type. Together, these features help identify and compare rhythmic molecular species across diverse datasets and biological contexts (Fig. 1B).

### 3.2 Dataset repository

CircadiOmics provides a large collection of circadian omic datasets through its dataset repository, where users can browse available datasets along with detailed metadata describing their experimental design and key characteristics. In addition to the existing keyword-based search, new filtering panels allow users to refine datasets by species, tissue/organ, and experiment type. The metadata have also been expanded to include information, where applicable, such as assay type, platform, molecule, genome build, aligner, quantifier, and expression unit. For publicly available datasets, the repository further provides a reference to the original study, allowing users to trace each dataset back to its original public repository and obtain the source data. These datasets and their corresponding BIO_CYCLE results are also available to users upon request. Together, these features enable efficient retrieval and analysis of circadian omic data across diverse biological studies.

### 3.3 BIO_CYCLE portal

The BIO_CYCLE portal enables users to upload and analyze their own time series omic datasets for rhythmicity detection. For each uploaded dataset, BIO_CYCLE computes and reports molecular-level periodicity statistics at 24-, 12-, and 8-hour periods. In the updated release, dedicated GPU resources enable this analysis to typically run in under one minute when a pre-trained DNN model is available and within five minutes otherwise. Once the analysis is complete, summary visualizations, including histograms showing the distributions of rhythmicity parameters across the dataset, are made available through the portal. Users can further explore the results by querying individual molecular species, applying user-defined *P*-value thresholds, and interactively visualizing expression trajectories by molecular identifier (Fig. S3, available as supplementary data at *Bioinformatics* online). An example dataset is provided to illustrate the required input format, and detailed documentation is available through the CircadiOmics help section. BIO_CYCLE is also available for download for local analysis, with source code, pre-trained models, and documentation accessible through CircadiOmics and GitHub.

### 3.4 Metabolic Atlas

The Metabolic Atlas section within CircadiOmics is available for metabolomics datasets and enables the generation and visualization of interactive metabolic networks derived from KEGG pathway annotations (Kanehisa and Goto 2000). Users can select a metabolomics dataset and a metabolite of interest and generate networks based on multiple criteria, including metabolites that oscillate in-phase with the selected metabolite or metabolites participating in the same metabolic pathways. In total, six network construction options are available, enabling flexible exploration of rhythmic metabolic relationships. The resulting networks are interactive and can be filtered using BIO_CYCLE rhythmicity statistics. These features help users focus on statistically supported rhythmic metabolites and examine pathway-level connections among them.

### 3.5 Single-cell module

A major addition in this CircadiOmics update is a new module for single-cell circadian transcriptomic analysis. The current release includes 15 single-cell transcriptomic datasets spanning three species and six tissues/organs, establishing a resource for circadian analysis at single-cell resolution. In studies without existing cell-type labels, annotations are assigned using Scanpy (Wolf *et al*. 2018) in conjunction with appropriate reference atlases. BIO_CYCLE rhythmicity statistics are then computed separately for each annotated cell population in each dataset. Details of the cell-type annotation, preprocessing, data filtering, and expression-value resampling are provided in Supplementary Note 2, available as supplementary data at *Bioinformatics* online. Users can explore each dataset by selecting a cell population and interactively visualizing gene expression trajectories within that population. By extending circadian analysis beyond bulk-tissue averages, this framework enables investigation of cell-type-specific rhythmic expression profiles and comparison of rhythmic gene regulation across cell populations within and across tissues, species, and experimental conditions.

### 3.6 Applications

CircadiOmics has diverse applications in circadian rhythm research. It enables users to explore a large collection of datasets, perform comparative analyses across circadian experiments, and analyze their own datasets using BIO_CYCLE. These capabilities have made CircadiOmics a valuable resource for biological discovery and hypothesis generation, supporting studies published in high-impact journals. For example, Lee *et al.* examined circadian gene-expression patterns across multiple primate brain regions using baboon transcriptomic datasets available through the repository. They found that shared rhythmic gene-expression programs were aligned more strongly with functional connectivity than with anatomical proximity, highlighting a circuit-level organization of circadian regulation in the primate brain (Lee *et al*. 2023). Similarly, Chen *et al.* examined cilia-associated genes across six mouse brain regions and found that rhythmic ciliary transcripts showed region-specific patterns and were substantially altered across physiological and pathological conditions, including cocaine treatment, dopamine D2 receptor deletion, high-fat diet, and epilepsy (Chen *et al*. 2024). CircadiOmics has also supported targeted gene-level analysis through its search functionality. For example, in the study by Villanueva-Carmona *et al.* the platform was used to identify a circadian expression profile for *SUCNR1* in primate datasets, particularly baboons, as part of an investigation of *SUCNR1* in circadian and metabolic regulation (Villanueva-Carmona *et al*. 2023). In addition, the BIO_CYCLE portal in CircadiOmics has supported rhythmicity analysis of time series datasets, including studies of astrocytic insulin signaling in the sex-specific integration of feeding-time and lighting cues (González-Vila *et al*. 2023), chronic intermittent hypoxia–associated remodeling of hepatic and systemic metabolic rhythms (Montellier *et al*. 2026), and tuning of transcriptional rhythms in skeletal muscle to regulate mitochondrial function (Sica *et al*. 2026). Together, these examples illustrate the broad applicability of CircadiOmics in circadian rhythm research.

## 4 Discussion

The new release of CircadiOmics substantially expands the scale and scope of the platform for circadian omic research. Through updates to BIO_CYCLE and continued growth of the dataset repository, CircadiOmics supports large-scale investigation of circadian regulation across diverse biological contexts. The addition of a single-cell module further broadens the platform beyond bulk-level studies and establishes a foundation for investigating cell-type-specific circadian regulation. Collectively, these advances strengthen CircadiOmics as a resource for the analysis and exploration of circadian omic data. By enabling comparative analyses of molecular rhythms across species, tissues, and experimental conditions, CircadiOmics advances the study of circadian regulation with broad implications for biology and medicine.

## 5 Limitations and future work

CircadiOmics includes datasets from different studies, which may differ in experimental protocols, sequencing platforms, normalization procedures, and quantification methods. Therefore, cross-study comparisons of molecular measurements should take these differences into account. In addition, because relatively few circadian single-cell datasets are currently available, the single-cell module has limited coverage. As additional circadian single-cell datasets become available, future updates to CircadiOmics will expand its coverage and diversity.

## Supplementary Material

btag716_Supplementary_Data

## Data Availability

The BIO_CYCLE source code is available on GitHub: https://github.com/BaldiLab/BIO_CYCLE. An archival snapshot is deposited on Zenodo (DOI: https://doi.org/10.5281/zenodo.22108329).
