# Supplementary material for: CircadiOmics: bioinformatics platform for circadian rhythms
Source: Bioinformatics. 2026 Sep 28;42(10):btag716. doi: 10.1093/bioinformatics/btag716 (PMC13637602; doi:10.1093/bioinformatics/btag716)
Supplement: Supplementary file 1 [file btag716_supplementary_data.pdf]

Supplementary Materials

CircadiOmics: bioinformatics platform for circadian rhythms

Kousha Changizi Ashtiani and Pierre Baldi

Supplementary Notes

1 BIO_CYCLE software updates

1.1 Updated p-value estimation

BIO_CYCLE estimates periodicity significance for each molecular trajectory using two statistics: a periodicity score derived from the deep neural network (DNN), denoted α, and a normalized measure of variability among biological replicates, denoted β. A p-value is calculated separately for each statistic, and the two p-values are then combined to obtain the final BIO_CYCLE p-value.

To calculate the replicate-variability statistic, consider a trajectory containing T time points, with R_t replicates at time point t. The mean expression at each time point is calculated as

$$\mu_t = \frac{1}{R_t}\sum_{r=1}^{R_t} x_t^r, \tag{1}$$

where x_t^r is the expression value of replicate r at time point t. The standard deviation among replicates at each time point is quantified as

$$\sigma_t = \sqrt{\frac{1}{R_t - 1}\sum_{r=1}^{R_t}(x_t^r - \mu_t)^2}, \tag{2}$$

and the average replicate standard deviation across all time points is calculated as

$$\sigma_{\text{rep}} = \frac{1}{T}\sum_{t=1}^{T}\sigma_t. \tag{3}$$

Because the magnitude of σ_{rep} depends in part on the amplitude of the molecular trajectory, it is normalized by the standard deviation of the mean trajectory. The mean of the mean trajectory is

$$\mu_{\text{traj}} = \frac{1}{T}\sum_{t=1}^{T}\mu_t, \tag{4}$$

and the standard deviation of the mean trajectory is

$$\sigma_{\text{traj}} = \sqrt{\frac{1}{T-1}\sum_{t=1}^{T}(\mu_t - \mu_{\text{traj}})^2}. \tag{5}$$

The normalized replicate-variability statistic is then defined as

$$\beta = \frac{\sigma_{\text{rep}}}{\sigma_{\text{traj}}}. \tag{6}$$

In implementation, BIO_CYCLE handles edge cases in which the standard deviation of the mean trajectory approaches zero or a time point contains only one replicate, for which replicate

variability cannot be estimated. When the standard deviation of the mean trajectory approaches zero, a small numerical offset is used to stabilize the denominator, while time points containing only a single replicate are not used to estimate replicate variability. If the time series input contains only one replicate at each time point, the replicate-variability component is not used to penalize the final periodicity significance during p-value combination.

Lower values of β indicate greater agreement among biological replicates relative to the temporal variation of the mean trajectory, whereas larger values indicate greater within-time-point variability.

To estimate the replicate-variability p-value, BIO_CYCLE constructs a sampling-specific null distribution for the normalized replicate-variability statistic, β. A gamma distribution is first fitted to the β values of established core clock genes across selected replicated circadian datasets to estimate the alternative distribution, $P(\beta \mid H_1)$. Based on previous studies reporting that approximately 10% of transcripts exhibit circadian rhythmicity in representative circadian experiments (Panda et al., 2002; Storch et al., 2002), BIO_CYCLE uses a fixed 10% rhythmic fraction as a modeling assumption for this estimation procedure, with the alternative component assumed to account for 10% of the observed β distribution. This proportion is not intended to represent a universal fraction of rhythmic transcripts across all datasets. A second gamma distribution is then fitted to the residual component not explained by $P(\beta \mid H_1)$, providing a reference null distribution (Supplementary Figure S4). BIO_CYCLE next uses this reference distribution to calibrate the Gaussian noise parameter, σ_n, which controls replicate variability in a replicated aperiodic-data model, selecting the value of σ_n that maximizes the likelihood of generating trajectories with β values consistent with the reference null distribution. The calibrated model is then used to generate the sampling-specific null distribution $P(\beta \mid H_0)$ for the dataset being analyzed. Because smaller values of β indicate greater agreement among biological replicates, the replicate-variability p-value, p_β, is calculated from the lower tail of this null distribution.

A separate p-value, p_α, is obtained from the DNN-derived periodicity score. BIO_CYCLE provides several methods for combining p-values, including Fisher's method (Fisher, 1925), Stouffer's method (Stouffer et al., 1949), Edgington's method (Edgington, 1972), and Friston's method (Friston et al., 1999). CircadiOmics uses Friston's method for the rhythmicity statistics reported for datasets in the repository. Under this method, the two component p-values, p_α and p_β, are combined as

$$p = \left[\max\left(p_\alpha, p_\beta\right)\right]^2 . \tag{7}$$

Because the larger of the two component p-values determines the combination, both p_α and p_β must be small for the resulting combined p-value to be small. Thus, a trajectory must exhibit both a strong periodicity signal and low replicate variability. This helps reduce false-positive rhythmicity calls for trajectories that exhibit an apparent periodic pattern but substantial variability among biological replicates.

1.2 Handling of missing time points

The previous implementation of BIO_CYCLE could accommodate missing individual replicates but could not directly process time points for which all replicate measurements were absent. To address this limitation, BIO_CYCLE takes advantage of the larger DNN architecture with residual blocks (Supplementary Figure S2) and simulates missing time points during training by randomly masking up to 20% of the input time points and setting their values to 0. The input trajectory is additionally concatenated with an indicator vector in which a value of 1 denotes an observed time point and a value of 0 denotes a missing time point. At inference, completely missing time points are likewise represented by setting their input values to 0 and the corresponding entries in the indicator vector to 0. This representation allows the DNN to dis-

tinguish an observed value of 0 from a completely missing time point and enables BIO_CYCLE to analyze trajectories with incomplete temporal sampling.

2 Single-cell data processing and rhythmicity analysis

For datasets consisting of pre-sorted cell populations, CircadiOmics uses the cell-type groupings provided in the original study metadata. For the remaining datasets, cell-type labels are assigned by reference-based label transfer using a tissue-appropriate publicly available reference atlas, with links to the corresponding atlases provided for each dataset. The query and reference matrices are processed identically using total-count normalization to 10,000 counts per cell (CP10K), followed by a log1p transformation. The top 2,000 highly variable genes are selected from the reference dataset and used to construct a shared 50-component principal-component space for the query and reference datasets. Cell-type labels are then transferred by k-nearest-neighbor classification with $k = 15$ in this shared space.

Following cell-type annotation, the raw count matrix is retrieved from the stored counts layer, and quality filtering is performed independently within each cell type. At the cell level, cells with fewer than 200 detected genes or fewer than 500 total counts are excluded. At the gene level, genes detected in fewer than three cells or with fewer than five total counts across the remaining cells of that cell type are removed. After filtering, expression values are normalized to CP10K within each cell, and the resulting matrix is used for downstream analysis.

For each cell type, the resulting expression matrix is subjected to a parametric resampling procedure to represent within-time-point variability, rather than being collapsed into a single pseudobulk value at each time point. For each gene and time point independently, a zero-hurdle Gaussian model is fitted to the CP10K expression values across cells of a given cell type. The model estimates the fraction of cells with zero expression (π), together with the mean (μ) and standard deviation (σ) of the log1p-transformed non-zero CP10K values. Ten synthetic replicates are then generated for each time point. Each replicate is assigned a value of zero with probability π; otherwise, a value is sampled from $\text{Normal}(\mu, \sigma^2)$ on the log scale, transformed back to the CP10K scale using the inverse log1p transformation, $\exp(x) - 1$, and constrained to be non-negative.

As part of quality control, genes are retained for rhythmicity analysis only when they exhibit non-zero expression across a minimum number of time points. The threshold is five time points for densely sampled circadian series containing at least 12 time points and two time points for the remaining datasets. A fixed random seed is used for resampling to ensure reproducibility. The resulting synthetic-replicate matrix, with columns corresponding to individual time-point replicates, is then analyzed using BIO_CYCLE, allowing rhythmicity inference to incorporate both temporal expression patterns and estimated within-time-point variability.

Because comprehensive ground truth sets of rhythmic and non-rhythmic genes are not available for these single-cell datasets, the resulting rhythmicity estimates are evaluated separately for each dataset by examining the overall distribution of BIO_CYCLE p-values across genes and the rhythmic expression patterns of established core clock genes, including *Arntl* (*Bmal1*), *Clock*, *Per1*, *Per2*, *Per3*, *Cry1*, *Cry2*, *Nr1d1*, and *Nr1d2*, to assess the biological plausibility of the resulting estimates.

Supplementary Tables

Table S1. Tissue/organ groups and associated tissues represented in CircadiOmics.

Tissue/organ group	Associated tissues
**Adipose**	Brown Adipose Tissue; IWAT; Mesenteric White Adipose Tissue; Omental Fat; White Adipose Tissue
**Bladder**	Bladder
**Blood**	Blood; Serum
**Bone Marrow**	Bone Marrow
**Brain**	Amygdala; Arcuate Nucleus; Brainstem; Central Forebrain; Central Nucleus of the Amygdala; Cerebellum; Choroid Plexus; Cortex; Dorsal Vagal Complex; Dorsomedial Hypothalamus; Forebrain; Habenula; Head; Hippocampus; Hypothalamus; Lateral Globus Pallidus; Lateral Hypothalamus; Lateral Hypothalamus Caudal; Lateral Hypothalamus Rostral; Left Prefrontal Cortex; Mammillary Body; Medial Globus Pallidus; Olfactory Bulb; Paraventricular Nucleus; Paraventricular Nucleus Thalamus; Periventricular Zone; Pons; Prefrontal Cortex; Preoptic Area; Putamen; Substantia Nigra; Suprachiasmatic Nucleus; Supraoptic Nucleus; Thalamus; Ventral Hippocampus; Ventral Striatum; Ventromedial Hypothalamus; Visual Cortex; Whole Brain
**Breast**	Breast
**Cells**	Bone Marrow Derived Macrophages; Breast Cancer Epithelial Cell Line; Cells; Embryonic Fibroblasts; Erythroid Progenitor Cells; Fibroblast; Pancreatic Cancer Cells; Pancreatic Cancer Organoid
**Digestive**	Antrum; Ascending Colon; Cecum; Colon; Descending Colon; Digestive; Distal Colon; Duodenum; Ileum; Intestine; Oesophagus; Rumen Epithelium Cells; Stomach Fundus
**Embryo**	Embryo
**Eye**	Choroid; Cornea; Iris; Optic Nerve Head; Retina; Retinal Pigment Epithelium
**Fat Body**	Fat Body
**Fungus**	Fungus; Mycelia
**Glands**	Adrenal Cortex; Adrenal Gland; Adrenal Medulla; Glands; Pancreas; Pineal Gland; Pituitary; Salivary Gland; Thyroid
**Heart**	Aorta; Heart; Ventricles
**Kidney**	Kidney; Kidney Cortex; Kidney Medulla
**Larva**	Larva
**Liver**	Humanized Liver; Liver
**Lung**	Lung
**Lymph Nodes**	Axillary Lymph Nodes; Mesenteric Lymph Nodes
**Muscle**	Extensor Digitorum Longus; Gastrocnemius; Muscle; Muscle Abdominal; Quadriceps; Skeletal Muscle; Smooth Muscle; Soleus; Tibialis Anterior
**Nervous**	Neurons; Nodose Ganglion; Peripheral Olfactory

Continued on next page

Tissue/organ group	Associated tissues
**Parasite**	Parasite
**Plant**	Whole Seedling
**Reproductive**	Ovary; Prostate; Sertoli Cells; Sperm; Testis; Uterus
**Skin**	Epidermis; Foreskin; Skin
**Spleen**	Spleen
**Tendon**	Extensor Digitorum Longus Tendon; Gastrocnemius Tendon; Tibialis Anterior Tendon
**Thorax**	Thorax
**Tumor**	CcRCC; Humanized Hepatocellular Carcinoma; Lung Tumor
**Whole Body**	Whole Body

Note: Tissue and organ labels were curated from the source studies and harmonized into broader tissue/organ groups to support consistent search, visualization, and cross-study comparison in CircadiOmics. Source labels referring to specific anatomical subregions or related tissues were assigned to the most biologically appropriate higher-level category. For example, brain regions such as the hippocampus, hypothalamus, and suprachiasmatic nucleus were grouped under Brain; gastrointestinal tissues such as the colon, ileum, and duodenum were grouped under Digestive; and reproductive tissues such as ovary, testis, and uterus were grouped under Reproductive. When the source label already represented a distinct tissue or sample-source category, the original label was retained, as in Spleen and Fat Body.

Table S2. Comparison of CircadiOmics with other circadian repositories.

Source	Datasets	Species	Tissues
CircadiOmics	704	18	>100
CircaDB	43	2	15
CirGRDB	96	2	37
CircaKB	226	15	54
RhythmicDB	87	19	N/R[1]

[1] Not reported.

Repository statistics were accessed in August 2026.

Table S3. Experiment types and associated conditions represented in CircadiOmics.

Experiment type	Conditions
**Age**	Aged; Early-Embryonic; Late-Embryonic; Old; Young
**Cancer**	Ameloblastoma; Breast Cancer; Kidney Cancer; Liver Cancer; Lung Cancer; Pancreatic Cancer
**Control**	Control
**Diet**	24-hr Fasting; Ad Libitum; Caloric Restriction; Dark-Phase Feeding; Grain-Derived Volatile Fatty Acid Treatment; Hay-Derived Volatile Fatty Acid Treatment; High-Calorie Diet; High-Fat Diet; High-Fat Diet Recovery; High-Salt Diet; Ketogenic Diet; Leucine-Devoid Diet; Light-Phase Feeding; Low-Calorie Diet; Low-Fat Diet; Low-Salt Diet; Normal Chow; Reverse-Phase Feeding; Time-Restricted Feeding; Tryptophan-Modified Diet; Unanticipated Fasting
**Disease**	*Brugia malayi* Infection; Epilepsy; Hepatitis C Virus Infection; Influenza Infection; Myopia; Nonalcoholic Steatohepatitis
**Drug Treatment**	Alcohol Exposure; Cocaine Exposure; Epigenetic-Regulator Inhibition; Oncogenic Pathway Inhibition
**Energy Status**	Energy Deficient; Energy Sufficient
**Exercise**	Evening Exercise; Evening Sedentary; High Exercise; Low Exercise; Morning Exercise; Morning Sedentary; No Exercise; Treadmill Exercise
**Genetic Perturbation**	Deletion; Knockdown; Metabolic Regulator Inhibition; Overexpression; Reprogramming
**Immune Cell Depletion**	Neutrophil Depletion
**Immune Perturbation**	IL-10 Receptor Blockade
**Inflammation**	Lipopolysaccharide Treatment
**Injury**	Retinal Lesion; Traumatic Brain Injury
**Knockout**	Core Clock Knockout; Cre-Negative Control; Dopamine Signaling Knockout; Epigenetic-Regulator Knockout; Hypoxia-Response Knockout; Metabolic-Sensing Knockout; miRNA Regulation Knockout; Neurodevelopmental Signaling Knockout; Nuclear Receptor Knockout; Rescue; RNA Editing Knockout; Transcription Factor Knockout
**Light-Dark**	Chronic Jet Lag; Constant Darkness; Extended Light; Phase Shift
**Microbiome**	Germ-Free
**Mutation**	Core Clock Mutation; *Dmsn1* Mutant
**Oxygen Level**	Acute Hypoxia; Intermittent Hypoxia; Normoxia
**Sex**	Female; Male
**Sleep**	Acute Sleep Deprivation; Circadian Misalignment; Sleep Deprivation; Sleep Recovery
**Temperature**	18°C; 25°C; 27°C; 29°C; 32°C; 37°C

Supplementary Figures

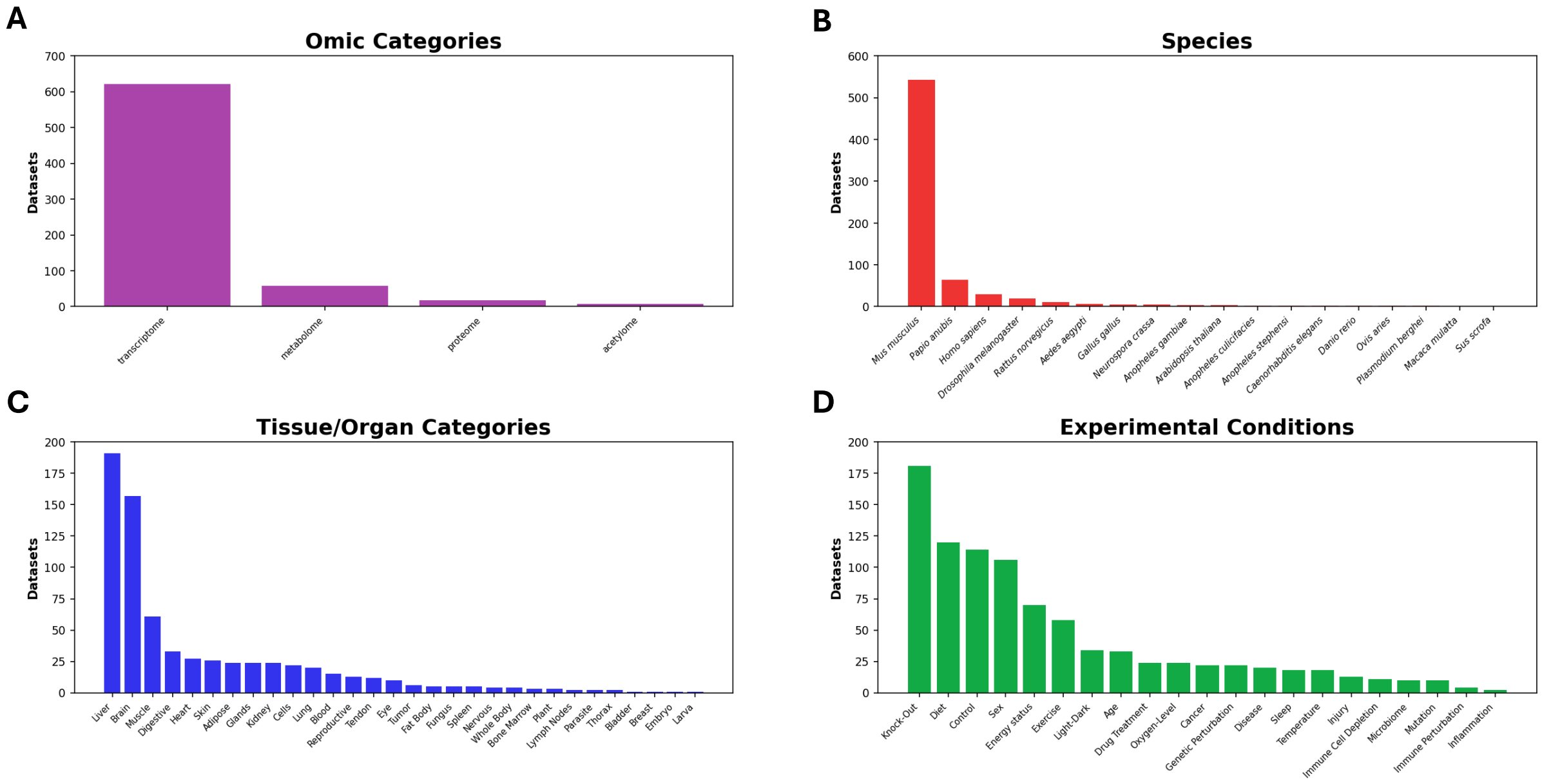

Figure S1. Diversity of omic data types, species, tissue/organ categories, and experimental conditions in CircadiOmics.

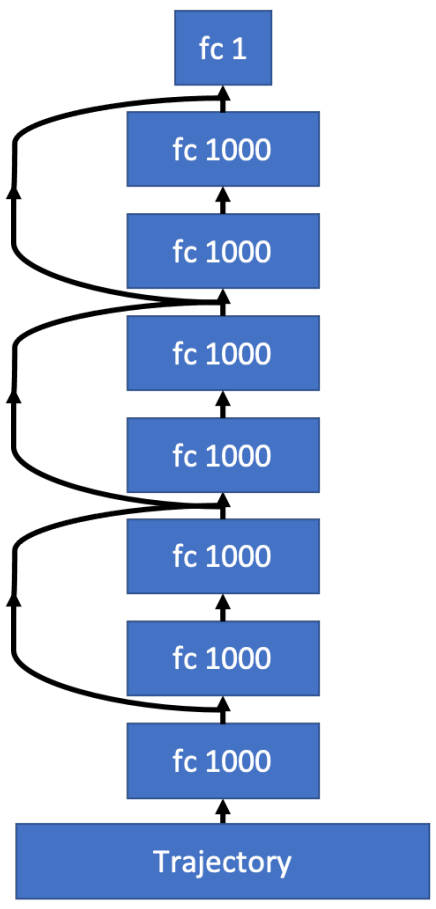

Figure S2. Visualization of the deep neural network architecture used by BIO_CYCLE to detect periodicity and estimate period.

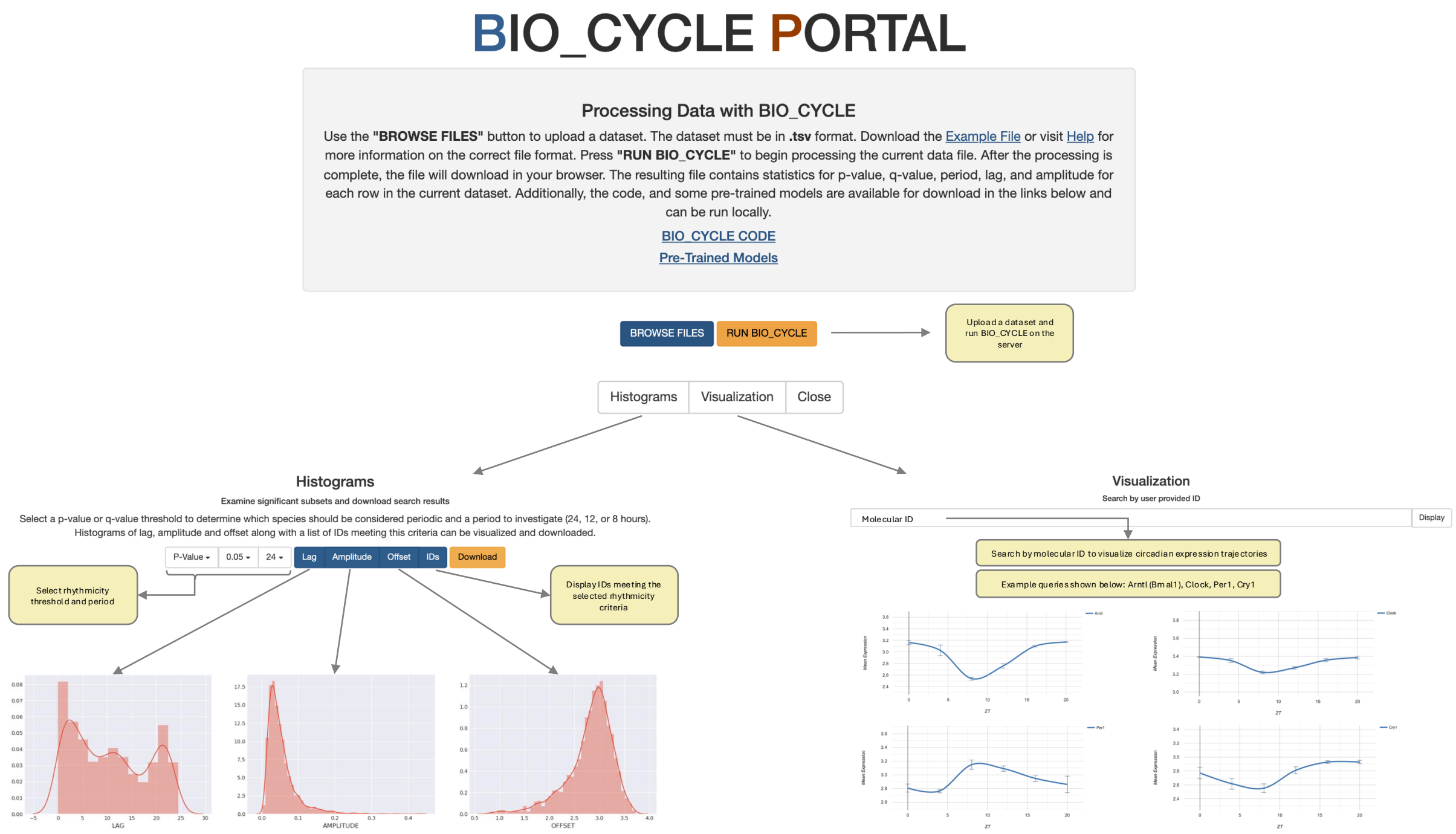

Figure S3. Overview of the BIO_CYCLE portal, where users can explore rhythmicity statistics for uploaded datasets and visualize circadian expression trajectories for individual molecular species.

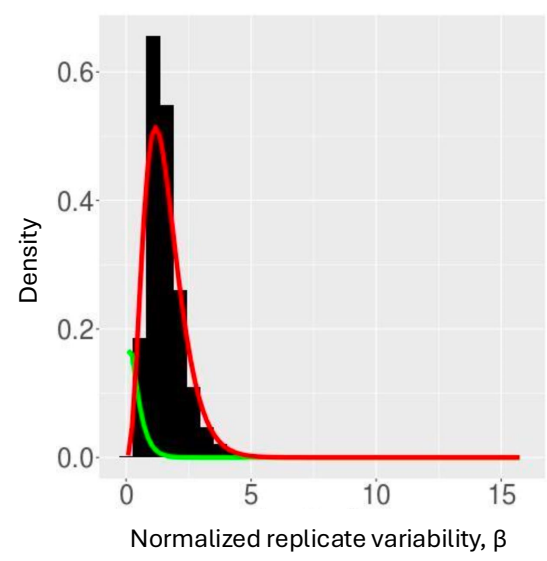

Figure S4. Distribution of the normalized replicate-variability statistic β, with fitted gamma distributions representing the reference null distribution (red) and the alternative distribution, $P(\beta \mid H_1)$ (green).

Supplementary References

Edgington ES. An additive method for combining probability values from independent experiments. *The Journal of Psychology*, 80(2):351–363, 1972. doi: 10.1080/00223980.1972.9924813.

Fisher RA. *Statistical Methods for Research Workers*. Oliver and Boyd, Edinburgh, 1925.

Friston KJ, Holmes AP, Price CJ, et al. Multisubject fMRI studies and conjunction analyses. *NeuroImage*, 10(4):385–396, 1999. doi: 10.1006/nimg.1999.0484.

Panda S, Antoch MP, Miller BH, et al. Coordinated transcription of key pathways in the mouse by the circadian clock. *Cell*, 109:307–320, 2002.

Storch KF, Lipan O, Leykin I, et al. Extensive and divergent circadian gene expression in liver and heart. *Nature*, 417:78–83, 2002.

Stouffer SA, Suchman EA, DeVinney LC, et al. *The American Soldier: Adjustment During Army Life*, volume 1 of *Studies in Social Psychology in World War II*. Princeton University Press, Princeton, NJ, 1949.
